# Enhanced neuroinflammation and pain hypersensitivity after peripheral nerve injury in rats expressing mutated superoxide dismutase 1

**DOI:** 10.1186/1742-2094-8-33

**Published:** 2011-04-13

**Authors:** Julie V Berger, Ronald Deumens, Stéphanie Goursaud, Sabrina Schäfer, Patricia Lavand'homme, Elbert A Joosten, Emmanuel Hermans

**Affiliations:** 1 Group of Neuropharmacology, Institute of Neuroscience, Université catholique de Louvain, Brussels, Belgium; 2 Department of Anesthesiology, Maastricht University Medical Centre, Maastricht, The Netherlands; 3 Department of Anesthesiology, Université catholique de Louvain, St. Luc Hospital UCL Medical School, Brussels, Belgium

## Abstract

**Background:**

Neuroinflammation and nitroxidative stress are implicated in the pathophysiology of neuropathic pain. In view of both processes, microglial and astroglial activation in the spinal dorsal horn play a predominant role. The present study investigated the severity of neuropathic pain and the degree of glial activation in an inflammatory- and nitroxidative-prone animal model.

**Methods:**

Transgenic rats expressing mutated superoxide dismutase 1 (hSOD1^G93A^) are classically used as a model for amyotrophic lateral sclerosis (ALS). Because of the associated inflammatory- and nitroxidative-prone properties, this model was used to study thermal and mechanical hypersensitivity following partial sciatic nerve ligation (PSNL). Next to pain hypersensitivity assessment, microglial and astroglial activation states were moreover characterized, as well as inflammatory marker gene expression and the glutamate clearance system.

**Results:**

PSNL induced thermal and mechanical hypersensitivity in both wild-type (WT) and transgenic rats. However, the degree of thermal hypersensitivity was found to be exacerbated in transgenic rats while mechanical hypersensitivity was only slightly and not significantly increased. Microglial Iba1 expression was found to be increased in the ipsilateral dorsal horn of the lumbar spinal cord after PSNL but such Iba1 up-regulation was enhanced in transgenic rats as compared WT rats, both at 3 days and at 21 days after injury. Moreover, mRNA levels of Nox2, a key enzyme in microglial activation, but also of pro-inflammatory markers (IL-1β and TLR4) were not modified in WT ligated rats at 21 days after PSNL as compared to WT sham group while transgenic ligated rats showed up-regulated gene expression of these 3 targets. On the other hand, the PSNL-induced increase in GFAP immunoreactivity spreading that was evidenced in WT rats was unexpectedly found to be attenuated in transgenic ligated rats. Finally, GLT-1 gene expression and uptake activity were shown to be similar between WT sham and WT ligated rats at 21 days after injury, while both parameters were significantly increased in the ipsilateral dorsal region of the lumbar spinal cord of hSOD1^G93A ^rats.

**Conclusions:**

Taken together, our findings show that exacerbated microglial activation and subsequent inflammatory and nitroxidative processes are associated with the severity of neuropathic pain symptoms.

## Background

Neuropathic pain is characterized by altered processing of nociceptive signals, mainly at the "first pain synapse" located at the level of the spinal dorsal horn. A prime role is commonly assigned to glial cells, surrounding these "first pain synapses" [1,2]. Particularly, microglia and astroglia have been shown to play pivotal roles in the onset and maintenance of pain hypersensitivity after peripheral nerve injury [3]. In healthy conditions, microglia ensure immunosurveillance of the central nervous system (CNS) [4] and astroglia, among others, regulate synaptic transmission through active clearance of ions and neurotransmitters [5]. Upon nerve injury however, glial cells become activated, as classically characterized by hypertrophied morphology, increased proliferation and up-regulation of specific markers, like ionized-calcium binding adaptor molecule 1 (Iba1) for microglia, or glial fibrillary acidic protein (GFAP) for astroglia [6,7]. Among many different glial activation states, some have been specifically related to neuropathic pain [8], characterized notably by up-regulation of pro-inflammatory proteins such as Toll-like receptor 4 (TLR4) [9] or NADPH oxidase 2 (Nox2) [10]. Moreover, the release of the pro-inflammatory cytokine interleukin 1β (IL-1β) was also found to contribute to pain hypersensitivity [11]. In addition, activation of astrocytes may interfere with the capacity of these cells to buffer the excitatory transmitter glutamate, through altered expression and/or function of astroglial glutamate transporters, GLAST and GLT-1, thereby affecting the pain synapse [12-14].

Although glial activation, and more specifically subsequent neuroinflammation and nitroxidative stress are known to strongly contribute to neuropathic pain [15-17], it remains unknown whether such parameters may influence the severity of pain symptoms. We herein addressed this issue by studying nerve injury induced-hypersensitivity in a rat model characterized by inflammatory- and nitroxidative-prone properties.

Classically used as a model for amyotrophic lateral sclerosis (ALS), transgenic rats expressing the mutated form of the human superoxide dismutase 1 (hSOD1^G93A ^[18] showing a toxic gain of function [19]) are characterized by excitotoxic insults and increased nitroxidative stress [20,21]. The high vulnerability of motor neurons to these pathological processes explains their progressive loss in adulthood, leading to paralysis and death from respiratory failure. Previous studies have indicated that cultured glial cells derived from the hSOD1^G93A ^model are more inclined to get activated, and once activated, the release of neuroactive mediators is amplified [22-24]. We therefore hypothesized that performing a peripheral nerve injury in hSOD1^G93A ^rats early before the onset of any symptom, will cause exacerbated glial responses and subsequently, an increased neuroinflammatory and nitroxidative environment in the spinal cord, with putative influence on the severity of neuropathic pain symptoms. All experiments were completed within the pre-symptomatic stage of transgenic animals, as no symptom of the disease (such as motor deficit or weight loss) can be detected before 150 days (unpublished observation, Goursaud S.). Both wild-type (WT) and transgenic rats aged 90 days underwent partial sciatic nerve ligation (PSNL) to study thermal and mechanical hypersensitivity up to 21 days after surgery. In addition, biochemical studies were performed to characterize microglial and astroglial activation states as well as inflammatory processes and regulation in the glutamate clearance system in the dorsal spinal cord.

## Methods

### General

All experiments were strictly performed respecting the European Community Council directive of 24 November 1986 (86-609/ECC) and the decree of 20 October 1987 (87-848/EEC). Transgenic Sprague-Dawley rats expressing hSOD1^G93A ^(multiple copies of the transgene, [25]) were kindly provided by Dr R. Pochet (Université Libre de Bruxelles, Belgium). Noteworthy and as commonly documented [26], the phenotype of the rats in our colony underwent a drift over multiple consecutive generations, leading to a postponed onset (150 days of age) and a slow disease progression. Animals were kept in controlled conditions (temperature, relative humidity, 12 h light/dark cycle) with constant access to food and water. To identify transgenic rats, tail biopsies from newborn rat pups (5-7 days old) were used, and the presence of the transgene was probed by PCR on genomic DNA extracts as previously described [27].

In this study, a total of 93 Sprague-Dawley female rats (females are characterized by a later ALS onset, [28]) were used, of which 46 were wild-type (WT) and 47 were transgenic (hSOD1^G93A ^positive, heterozygous). When the experiments started, all rats were aged 90 days and all experiments were completed within the pre-symptomatic stage.

First, the inflammatory-prone phenotype of the transgenic rats was investigated in our experimental paradigm. PSNL [29] or sham surgeries were performed on WT and transgenic rats that were sacrificed on post-operative day 3 or 21 to study microglial Iba1 gene expression. A second cohort of animals was used to monitor thermal and mechanical hypersensitivity for 21 days after surgery. After sacrifice, spinal tissues were analyzed for cellular and molecular changes related to a neuroactive environment (microglial and astroglial activation, expression of inflammatory markers) as well as for the study of glutamate transporters. All experiments were performed in a blinded manner, where the experimenters were unaware of experimental conditions.

### Animal surgeries

At 90 days of age, WT or hSOD1^G93A ^female rats were anesthetized with sevoflurane (8% for induction; 4% for maintenance; oxygen as carrier gas) and a PSNL was performed as previously described, with slight modifications [29]. Briefly, the right sciatic nerve was exposed at the mid-thigh level after isolation from the surrounding tissues. With a 6-0 suture, one third to one half of the nerve was tightly ligated at the site corresponding to the nerve trunk above its bifurcation in peroneal and tibial branches. Muscle and skin layers were finally closed with a 2-0 suture. Sham surgeries were performed by simply exposing the nerve before suturing the wound without performing the ligation.

### Behavioral testing

During the 2 weeks preceding surgeries, all animals used for behavioral analyses were habituated to the testing procedures.

To examine thermal hyperalgesia (Hargreaves test), rats were placed in separate transparent boxes, for at least 15 min before starting measurements. A beam of radiant heat was applied to the mid plantar surface of rat's hind paws (Paw Thermal Stimulator, San Diego University, USA) and paw withdrawal latencies (PWL) were measured for the ipsilateral and the contralateral sides, with a cut-off fixed at 20 s to avoid tissue damage. For each time-point, the PWL of at least four testing trials, each separated by a 10 min interval, were averaged per rat and normalized by the rat's own average PWL before surgery (baseline).

To assess mechanical allodynia (von Frey hair filament tests), rats were placed in the same boxes, on a metal mesh floor for at least 15 min to let them habituate to the experimental environment. The previously described up-down method was used with minor modifications to determine the 50% paw withdrawal threshold (PWT, [30]). In total, 9 different von Frey filaments (Stoelting Co, Dale, USA) were selected, with bending force from 0.4 g to 26 g. The filaments were applied perpendicularly to the mid plantar surface of hind paws until bending, for 8 s. A response was considered as positive only if the rat showed paw withdrawal with clear signs of aversion against the stimulus (i.e. postural movement, licking of the paw, maintaining the paw in up-lift position).

One day before surgery, baseline values were obtained for each rat. Post-operative assessments of pain behavior were performed on day 1, 2, 3, 5, 7, 10, 14, 17 and 20 post-surgery.

### Fresh tissue dissection

Rats were euthanized with CO_2 _and decapitated. The spinal cord was rapidly isolated by hydro-extrusion and transferred to cooled phosphate-buffered saline (PBS) solution to carefully remove meninges. The lumbar enlargement (L3-L6) was isolated and ipsilateral and contralateral sides were separated using the ventral fissure as a reference. Subsequently, these halves were divided into dorsal and ventral parts using the central canal as a reference. For measurement of glutamate transporter activity (*synaptosomes*, see below), fresh tissue samples were used immediately. For quantitative PCR (qPCR) experiments, tissues were frozen in 1 mL TriPure isolation reagent (Roche, Mannheim, Germany) before further use.

### RNA extraction, reverse transcription and real-time qPCR

After thawing of samples, the ipsilateral dorsal quadrant of the lumbar spinal cord was mechanically dissociated in TriPure isolation reagent, followed by RNA extraction according to the manufacturer's protocol. Reverse transcription was carried out with 1 μg of RNA using the iScript cDNA synthesis kit (BioRad, Nazareth, Belgium), in a total volume of 20 μL. Real-time qPCR were finally performed to specifically amplify Iba1, glutamate transporters (GLAST and GLT-1), cytokines (IL-1β), TLR4, Nox2 and GAPDH (see Table 1). Reactions were carried out in a total volume of 25 μL, containing 2 ng of cDNA sample, 350 nM of both specific forward and reverse primers and the IQ SYBR^® ^Green supermix 1 × (BioRad, 100 mM KCl, 40 mM Tris-HCl pH 8.4, 0.4 mM each dNTP, 50 U/ml of iTaq DNA polymerase, 6 mM MgCl_2_, SYBR Green I, 20 nM fluorescein, stabilizers). The protocol consisted of 45 amplification cycles, each characterized by 15 s of denaturation at 95°C, 45 s of hybridization at 60°C and 15 s of elongation at 79°C. The measurements were performed using the iCycler IQ multicolor real-time PCR detection system, while the analysis of raw data was performed via the "post run data analysis" software provided by BioRad.

**Table 1:** Real-time qPCR primers (F: forward primer, R: reverse primer) and size of amplicon

Gene	Sequence	Amplicon (bp)
GAPDH	F: 5'- GTCTCCTGTGACTTCAACAG -3' R: 5'- AGTTGTCATTGAGAGCAATGC -3'	76
Iba1	F: 5'- CAGAATGATGCTGGGCAAG -3' R: 5'- CCTCCAATTAGGGCAACTCA -3'	127
GLAST	F: 5'- GATCGGAAACATGAAGGAGC -3' R: 5'- CAAGAAGAGGATGCCCAGAG -3'	121
GLT-1	F: 5'- GGTCAATGTAGTGGGCGATT -3' R: 5'- GGACTGCGTCTTGGTCATTT -3'	124
Nox2	F: 5'- CCTTTCCTGCATCTGGGTCTCC -3' R: 5'- CGCCCTTTGCCTCCATTCTC -3'	164
IL-1β	F: 5'- GGAAGGCAGTGTCACTCATTGTG -3' R: 5'- GGTCCTCATCCTGGAAGCTCC -3'	84
TLR4	F: 5'- GATTGCTCAGACATGGCAGTTTC -3' R: 5'- CACTCGAGGTAGGTGTTTCTGCTAA -3'	135

For quantification, a relative standard curve was generated for each targeted gene by using a cDNA template mix (combining all experimental samples) used at serial dilutions. After plotting the threshold values of amplifications versus the logarithm of the amount of cDNA added for the reaction, the relative amount of the targeted gene in each sample condition was calculated. Values were finally normalized against the relative expression of the housekeeping gene GAPDH.

### Measurement of D-[^3^H]-aspartate uptake activity in spinal cord synaptosomes

After dissection, fresh tissue was immediately used to prepare synaptosomes, as previously described [31], with few modifications. The ipsilateral and contralateral dorsal part of the lumbar (L3-L6) spinal cord were homogenized in 5 mL of ice-cold (4°C) 0.32 M sucrose solution by repeating 10 up-and-down movements in a pre-chilled Teflon glass homogenizer. The homogenates were then centrifuged at 1000 g for 10 min at 4°C, and only the supernatants, containing the synaptosomes, were collected and stored on ice, while the pellets were resuspended in 5 mL of fresh 0.32 M sucrose solution at 4°C. After 10 up-and-down movements, the homogenates were again centrifugated at 1000 g for 10 min at 4°C. Supernatants were pooled and finally centrifuged at 17500 g for 30 min at 4°C. After discarding the supernatants, the pellets, containing the synaptosomes vesicles, were homogenized with 10 up-and-down strokes in 500 μL of a Krebs-ringer buffer (120 mM NaCl, 4.8 mM KCl, 1.3 mM CaCl_2_, 1.2 mM MgSO_4_, 1.2 mM KH_2_PO_4_, 25 mM NaHCO_3_, 6 mM glucose, pH 7.6) at 4°C. Protein concentrations were determined for each sample with the Bradford method. To assess glutamate transporter activity, D-[^3^H]-aspartate (specific activity of 11.3 Ci/mmol, PerkinElmer NEN, Zaventem, Belgium) was used as substrate (as aspartate and glutamate have similar affinities for glutamate transporters), at a tracer concentration of 50 nM. In a total volume of 500 μL, 40 μg of protein (synaptosome preparation) were then incubated with the substrate for 10 min at 37°C. To evaluate the GLT-1-dependent uptake, the specific inhibitor dihydrokainic acid (DHK, 100 μM, Tocris, Bristol, UK) was added to the reaction. The suspension was immediately filtered through a GF/B glass fiber filter adapted 96-well plate (UniFilter GF/B, PerkinElmer), and washed three times with the Krebs-Ringer buffer at 4°C. Filters were then dried overnight at 37°C. Microscint 20 (45 μL, PerkinElmer) was then added to each well of the filter plate, and after 24 h, plates were counted with a Topcount^® ^Microplate scintillation and luminescence counter (PerkinElmer). Results are expressed as pmol of D-[^3^H]-aspartate transported per min and per mg of protein.

### Immunohistochemistry and quantification

On day 21 post-surgery, rats were anesthetized using ketamine (80 mg/kg) and xylazine (10 mg/kg), and then sacrificed by transcardiac perfusion with saline solution (NaCl 0.9%) at 37°C, followed by perfusion with paraformaldehyde solution (4% PFA in PBS buffer) at 4°C. The spinal cord was isolated via hydro-extrusion and stored overnight in the PFA solution for a post-fixation at 4°C. Cryopreservation was performed by incubation in a 15% sucrose solution (in PBS buffer) for 24 h at 4°C, followed by incubation in a 30% sucrose solution for 3 consecutive days at 4°C. The lumbar region of each spinal cord (L3-L6) was frozen using dry ice. The samples were finally stored at -80°C until cryosectioning. In total, 20 spinal cord samples were serially cut at -30°C to obtain twelve series of transverse 30 μm thick sections which were collected on ThermoScientific superfrost Plus glass slides (VWR International, Leuven, Belgium). Sections were stored at -20°C until further histological staining. After thawing and drying, every 12^th ^section was stained for Iba1 or GFAP. For Iba1, sections were washed 3 times (3 × 10 min) under gentle shaking, in Tris-buffered saline (TBS) (pH 7.6), before incubation in a blocking solution (5% normal donkey serum (NDS), 1% Triton X-100, in TBS pH 7.6) for 1 h at 4°C. The primary antibody against Iba1 (rabbit anti-rat Iba1, 1:1000, Wako Pure Chemical Ltd, Osaka, Japan), was diluted in a working solution containing 1% NDS and 1% Triton X-100 (in TBS, pH 7.4), for an overnight incubation at 4°C. The next day, after 3 washing steps (3 × 10 min) with TBS, sections were incubated with secondary antibody (donkey anti-rabbit-Alexa488 antibody, 1:100, Invitrogen, Merelbeke, Belgium) diluted in the same working solution, for 1 h at 4°C. After 3 washes in TBS (3 × 10 min), sections were embedded in 80% glycerol in PBS, and finally cover-slipped.

The protocol of GFAP staining was similar, except for the washing buffer (use of TBS-T: TBS containing 0.3% of Triton X-100), the absence of a blocking step, and the antibody incubation conditions (use of a TBS-T solution containing the primary antibody rabbit anti-rat GFAP 1:1000 from DAKO Diagnostics, Heverlee, Belgium, and the overnight incubation at room temperature). The secondary antibody (donkey anti-rabbit-Alexa488 antibody, 1:100, Invitrogen) was diluted in a TBS-T solution for 1 h at room temperature. Sections were finally embedded and cover-slipped.

Photo-micrographs of the ipsilateral and contralateral dorsal horns were taken using a fluorescent microscope (Olympus Ax70, Paes, Zoeterwoude, The Netherlands) with a 4x-objective and a grey-scale F-view cooled CCD camera. A total of 10 sections (L4-L5) per animal were analyzed using the cell profiler software CellP^® ^(Olympus). The dorsal horn was delineated and after background subtraction, Iba1 and GFAP stained dorsal horns were analyzed. The percentage of dorsal horn showing positive immunostaining, also referred as "area fraction" (AF) was calculated for each photo-micrographs and data were finally plotted in a graph.

### Statistical analyses

Data were expressed as means ± standard error of the mean (SEM) and statistical analysis were performed with GraphPad Prism version 3.02 (GraphPad software, CA, USA). To compare the 4 different groups, a one-way ANOVA followed by the Tukey post-hoc test for multiple comparisons were used. A repeated measures ANOVA followed by a Tukey post-hoc test was used to compare the severity of pain hypersensitivity between WT and hSOD1^G93A ^animals. Values of *p*< 0.05 were considered as statistically significant. Symbols * were used to compare WT ligated *vs *WT sham rats, symbols $ were used to compare transgenic ligated *vs *transgenic sham rats, and finally, symbols # were used to compare WT ligated *vs *transgenic ligated rats. Two symbols indicate a *p *value < 0.01 while 3 symbols represent a *p *value < 0.001.

## Results

### Up-regulation of Iba1 gene expression is enhanced in hSOD1^G93A ^rats at 3 and at 21 days after PSNL

**Figure 1:**
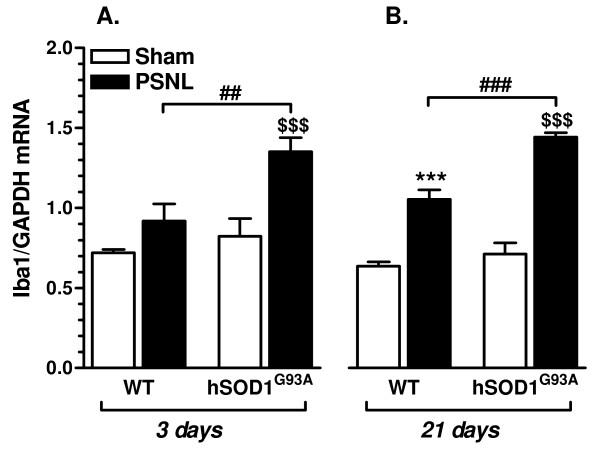
**Iba1 gene expression is enhanced in hSOD1^G93A ^rats at 3 and 21 days after PSNL**. Iba1 mRNA expression (normalized for GAPDH mRNA expression) is strongly increased in the ipsilateral dorsal quadrant of the lumbar spinal cord of transgenic rats at 3 days **(A) **and at 21 days **(B) **after PSNL, as compared to WT ligated animals, validating the inflammatory-prone phenotype of hSOD1^G93A ^rats after peripheral nerve injury. Each group included 5 animals, ## *p*< 0.01, $$$ *p*< 0.001, *** *p*< 0.001, ### *p*< 0.001 after ANOVA and Tukey post-hoc test.

Quantitative RT-qPCR measurements of the microglial marker Iba1 were performed on spinal cord samples in order to evaluate the inflammatory response after PSNL (Figure 1). Ipsilateral lumbar dorsal quadrants were dissected from WT or transgenic rats, at 3 days and at 21 days after surgery. At the early post-operative time-point (Figure 1A), WT ligated rats showed only a discrete increase in Iba1 mRNA levels as compared to their control group. In contrast, transgenic ligated rats showed a clear and statistically significant increase in gene expression of the microglial marker. The increase in Iba1 gene expression induced by PSNL was moreover significantly higher in transgenic rats as compared to WT animals. Importantly, mRNA expression of Iba1 did not differ between sham groups. At 21 days post surgery (Figure 1B), Iba1 gene expression appeared significantly increased in both ligated groups, but mRNA levels were clearly and significantly higher in samples from transgenic ligated rats when compared with WT ligated rats. Gene expression of the microglial marker was again found to be similar between both WT and transgenic sham groups.

### Rats expressing hSOD1^G93A ^show enhanced pain hypersensitivity after PSNL

**Figure 2:**
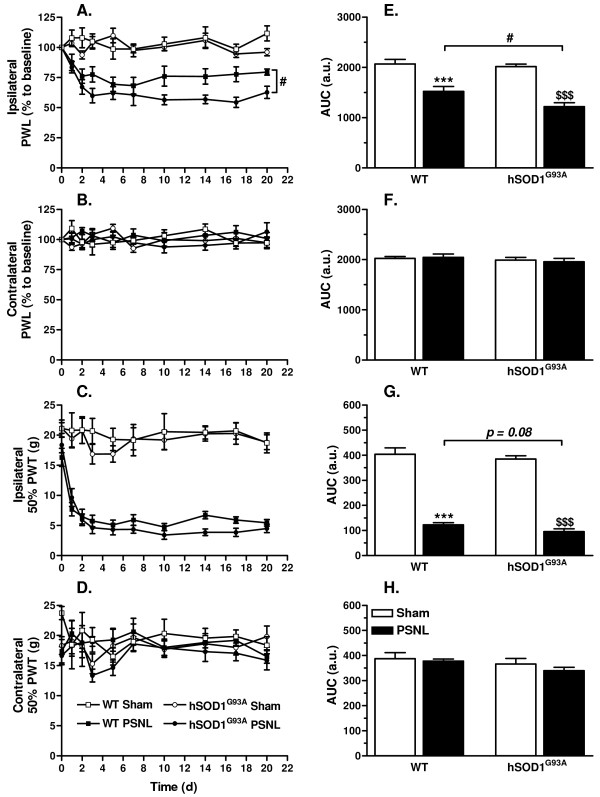
**Rats expressing hSOD1^G93A ^show enhanced pain hypersensitivity after PSNL**. Rats were tested for thermal and mechanical hypersensitivity for 21 days after surgery. Paw withdrawal latencies (PWL) of the ipsilateral **(A) **or contralateral **(B) **hind paws after thermal stimulation, expressed as a percentage of the baseline PWL. PSNL induced a decrease of PWL in both WT and hSOD1^G93A ^rats on the ipsilateral side, compared to their respective sham controls (statistics not shown). The ANOVA repeated measures revealed enhanced pain hypersensitivity for hSOD1^G93A ^rats compared to WT rats after PSNL. **(C,D) **50% paw withdrawal threshold (PWT) of the ipsilateral **(C) **or contralateral **(D) **hind paws after mechanical stimulation, expressed in grams. PSNL triggered mechanical hypersensitivity in both WT and transgenic rats (statistics not shown), but no statistical difference was evidenced between WT and transgenic rats after ligation. **(E-H) **Area under the curve (AUC) analysis of the corresponding graph confirmed the enhanced ipsilateral thermal hypersensitivity in transgenic rats compared to WT rats after PSNL (#*p *< 0.05), while mechanical hypersensitivity only tends to be amplified in transgenic rats after lesion (*p *= 0.08); ANOVA and Tukey post-hoc test. Each group included 6 to 8 animals.

Hypersensitivity to thermal and mechanical stimulation of hind paws was monitored during 21 days after sham or PSNL surgery, in both WT and transgenic rats (Figure 2). Noteworthy, uninjured WT and hSOD1^G93A ^rats showed similar baseline paw withdrawal latencies (PWL) and paw withdrawal thresholds (PWT). After PSNL however, a clear and significant decline in the withdrawal latency after thermal stimulation of ipsilateral hind paws was observed in ligated animals in comparison with their respective sham group (Figure 2A). Importantly, no difference in the PWL could be detected between both WT and transgenic sham groups throughout the period of thermal pain hypersensitivity assessment. The response was moreover unilateral as the withdrawal latency of contralateral paws was unaffected for both WT and hSOD1^G93A ^rats (Figure 2B). Interestingly, thermal pain hypersensitivity after PSNL was more pronounced for transgenic compared to WT animals. Area under the curve analysis confirmed the significantly enhanced ipsilateral thermal pain hypersensitivity for transgenic ligated rats as compared to WT ligated rats (Figure 2E), while contralateral values were unchanged (Figure 2F).

Consistent with data from thermal stimulation, mechanical pain hypersensitivity testing indicated that PSNL caused a robust decrease in the ipsilateral 50% PWT (Figure 2C) while no difference was observed on the contralateral side (Figure 2D). Both sham-operated groups showed similar 50% PWT until the endpoint of mechanical pain hypersensitivity testing. However, the ipsilateral 50% PWT did not differ between WT and transgenic rats after PSNL. Hence, the area under the curve analysis of the ipsilateral side (Figure 2G) indicated a trend (*p*< 0.08) towards a difference between WT ligated and hSOD1^G93A ^ligated animals. No changes were noticed in the area under the curve analysis for the contralateral side (Figure 2H).

### Rats expressing hSOD1^G93A ^show enhanced Iba1 protein up-regulation at 21 days after PSNL in the dorsal horn of the spinal cord

**Figure 3:**
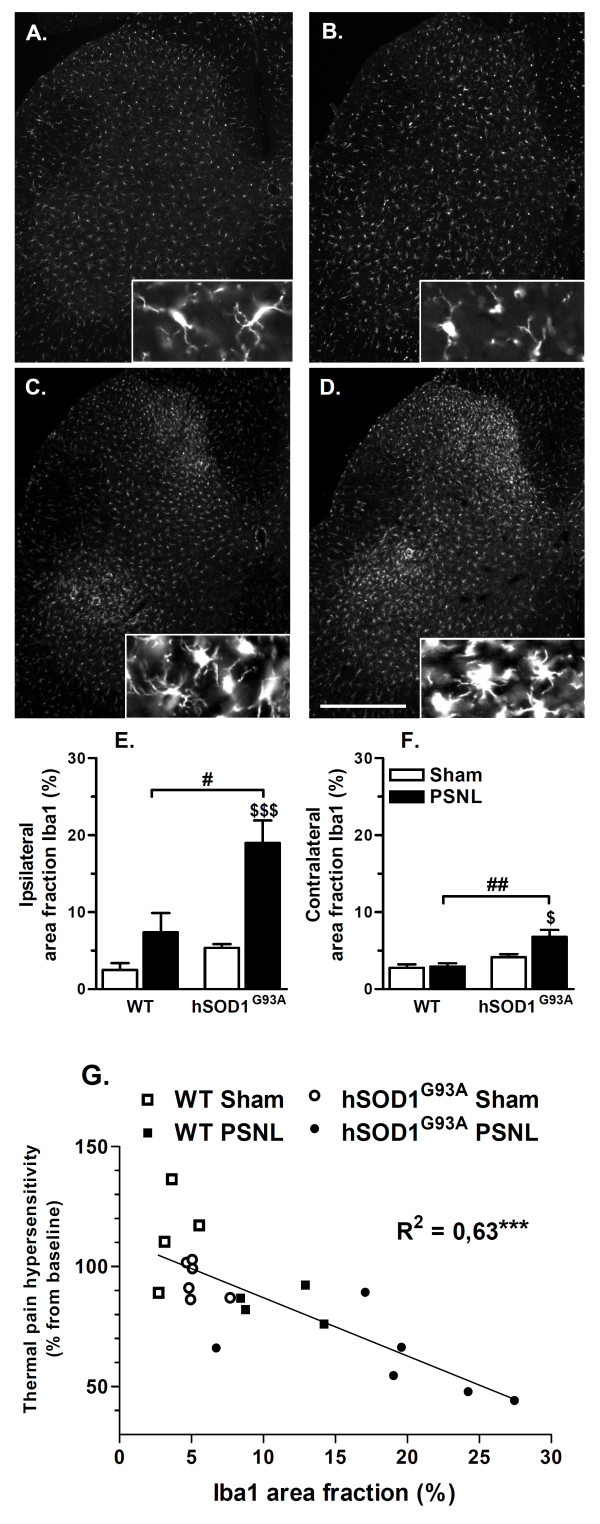
**Increased Iba1 protein up-regulation in transgenic rats at 21 days after PSNL**. **(A-D) **Immunohistological staining of the microglial marker Iba1 at 21 days after surgery, on the ipsilateral side of the lumbar spinal cord with a low and high magnification. **(A,B) **Microglia in both WT **(A) **and transgenic **(B) **sham groups showed a ramified morphology. After PSNL, microglia exhibited a hypertrophied morphology, an effect which was found to be stronger in transgenic rats **(D) **as compared to WT rats **(C) **(scale bar represents 500 μm). **(E,F) **Quantification of the percentage of the total dorsal horn showing positive Iba1 immunostaining. On the ipsilateral side **(E)**, Iba1 protein up-regulation was found to be stronger in transgenic compared to WT rats after PSNL ($$$ *p*< 0.001; # *p*< 0.05). The contralateral side **(F) **showed a modest Iba1 up-regulation only in transgenic rats at 21 days after PSNL ($ *p*< 0.05; ## *p*< 0.01). (**G**) The percentage of change in PWL from baseline on post-operative day 21 appeared to be correlated with the level of Iba1 up-regulation (R^2 ^= 0.63 and *p*< 0.001). WT groups included 4 animals whereas hSOD1^G93A ^groups included 6 animals; ANOVA and Tukey post-hoc test for statistical analyses.

Immunohistochemical analysis of Iba1 protein expression was performed on lumbar spinal cord sections from rats sacrificed at 21 days after surgery. In both WT and hSOD1^G93A ^sham-operated groups, microglia were characterized as small and ramified cells (Figure 3A and 3B). Similar observations were made in the contralateral dorsal horn of WT rats with sham or PSNL surgery (data not shown). In contrast, a marked increase in Iba1 immunoreactivity was evidenced in the ipsilateral dorsal horn of both WT and hSOD1^G93A ^ligated groups, where microglia were typically hypertrophied and less ramified (Figure 3C and 3D). To a lower extent, such hypertrophied microglia were also observed in the contralateral dorsal horn of the hSOD1^G93A ^rats with PSNL (data not shown). Quantitative analyses showed that the ipsilateral Iba1 area fraction was higher for rats with PSNL as compared to sham operated-rats. This difference was statistically significant for transgenic animals (*p*< 0.001) and showed a trend towards statistical significance for WT animals (Figure 3E). Overall, ipsilateral Iba1 protein expression associated with PSNL was found to be stronger in hSOD1^G93A ^rats as compared to WT animals (*p*< 0.05). Analysis of the contralateral dorsal horn demonstrated that PSNL significantly increased Iba1 area fraction in transgenic animals only (Figure 3F). Ipsilateral and contralateral Iba1 area fraction of the dorsal lumbar spinal horn were moreover found to be similar between both WT and transgenic sham groups. Finally, ipsilateral Iba1 area fraction values were interestingly found to be correlated (*p < 0.001*) with the percentage of change of the ipsilateral PWL from the baseline, on post-operative day 21 (Figure 3G). Noteworthy, as the PSNL damages both sensory and motor neuron processes in the sciatic nerve, increased Iba1 immunoreactivity was logically observed in the ipsilateral ventral horn of WT and transgenic ligated animals, though such responses were not further quantified.

### Rats expressing hSOD1^G93A ^show impaired GFAP protein up-regulation at 21 days after PSNL in the dorsal horn of the spinal cord

**Figure 4:**
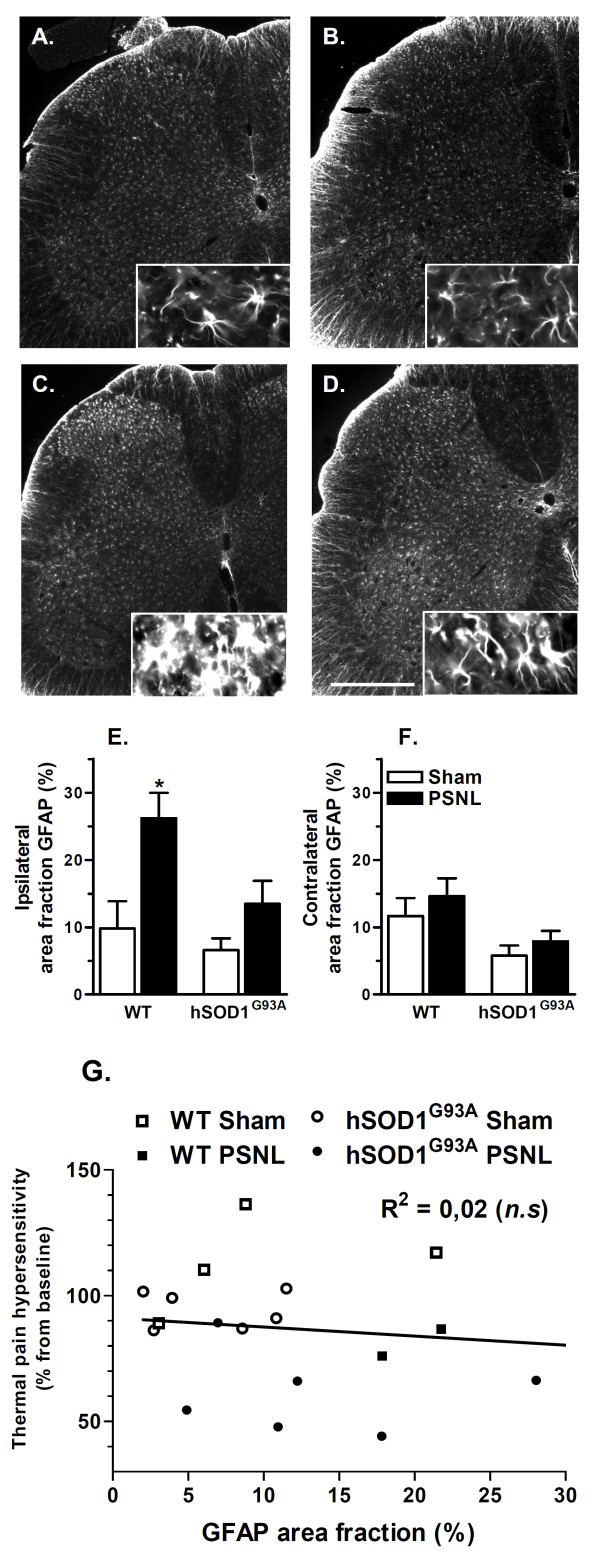
**GFAP protein up-regulation is attenuated in hSOD1^G93A ^rats at 21 days after PSNL**. **(A-D) **Immunohistological staining of the astroglial marker GFAP at 21 days after surgery, on the ipsilateral side of the lumbar spinal cord with a low and high magnification. **(A,B) **Astrocytes in the ipsilateral dorsal horn of WT **(A) **or transgenic **(B) **rats after sham operation showed a typical star-shaped morphology. At 21 days after PSNL, WT rats showed a strong astroglial response, characterized by cell hypertrophy and increased GFAP staining **(C)**. In transgenic rats, this astroglial response was clearly attenuated **(D) **(scale bar represents 500 μm). **(E,F) **Quantification of the percentage of the total dorsal horn showing positive GFAP immunostaining. GFAP protein up-regulation was validated on the ipsilateral side **(E) **only for WT ligated rats, while transgenic rats showed a modest but non-significant increase in GFAP expression after PSNL. The contralateral side **(F) **did not show any statistical changes in GFAP area fraction. (**G**) The percentage of change in PWL from baseline on post-operative day 21 did not correlate with the level of GFAP up-regulation (R^2 ^= 0.02). WT groups included 4 rats whereas hSOD1^G93A ^groups included 6 rats; ANOVA and Tukey post-hoc test for statistical analyses.

Immunodetection of GFAP expression was also performed on lumbar spinal cord sections in order to characterize the spreading of the astroglial response induced by PSNL in WT and hSOD1^G93A ^rats (Figure 4A-D). Three weeks after lesion, GFAP immunoreactivity was found to be similar in the ipsilateral dorsal horn of both sham groups (Figure 4A and 4B), while the signal was clearly increased only in WT rats after ligation. With respect to cell morphology, immunoreactive cells appeared hypertrophied only in WT injured animals (Figure 4C), while few changes were observed in hSOD1^G93A ^ligated rats (Figure 4D). Quantitative analysis of the area fraction of the ipsilateral dorsal horns from GFAP staining revealed significant increase following PSNL only for WT animals. For the transgenic model, GFAP area fraction was modestly but not significantly increased in comparison with sham littermates (Figure 4E). In the contralateral dorsal horn, no statistical changes could be detected for all animal groups (Figure 4F). Finally, no significant correlation was observed between ipsilateral GFAP area fraction and PWL on post-operative day 21 (Figure 4G). Here again, PSNL resulted in astroglial activation in the ipsilateral ventral horn of both WT and transgenic rats.

### Glutamate transporter expression and activity are increased after PSNL in hSOD1^G93A ^rats

**Figure 5:**
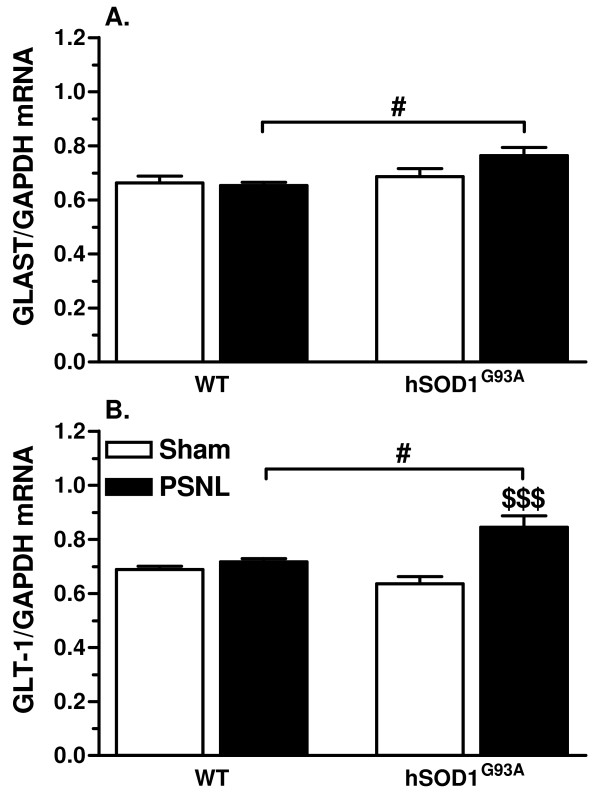
**hSOD1^G93A ^rats show higher mRNA levels of the glutamate transporters GLAST and GLT-1 after PSNL**. **(A) **mRNA expression of the glutamate transporter GLAST (normalized for GAPDH mRNA expression) is slightly increased in the ipsilateral dorsal horn of transgenic rats when compared to WT animals at 21 days after PSNL (#*p*< 0.05). **(B) **mRNA levels of the glutamate transporter GLT-1 after GAPDH normalization are higher in the ipsilateral dorsal horn of transgenic rats at 21 days after PSNL when compared to the sham control rats or to WT ligated rats ($$$ *p*< 0.001, # *p*< 0.05). Each group included 5 animals, ANOVA and Tukey post-hoc test for statistical analyses.

Changes in glial glutamate handling likely contribute to altered pain transmission in the spinal cord of animals with peripheral nerve injuries. Therefore, the expression and activity of the two key glial glutamate transporters were compared in samples from WT and transgenic animals after PSNL. Quantitative PCR revealed that no significant changes in GLAST and GLT-1 mRNA levels were detected at 21 days after lesion in the ipsilateral dorsal quadrant of the lumbar spinal cord of WT rats. In contrast, a discrete but significant increase in GLAST expression was observed when comparing the groups of ligated animals (Figure 5A). Such difference was even more pronounced for GLT-1, as statistical analysis revealed increased expression associated with PSNL in transgenic rats when compared either to transgenic sham littermates or to WT ligated rats (Figure 5B).

**Figure 6:**
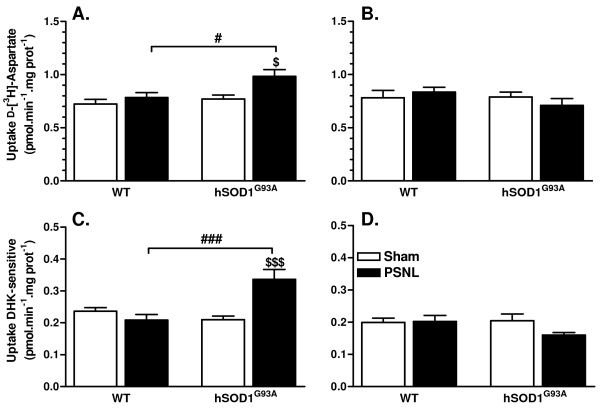
**hSOD1^G93A ^rats show increased glutamate uptake capacities after PSNL**. **(A, B) **Measure of the total uptake activity in the ipsilateral **(A) **and the contralateral **(B) **dorsal quadrant of the lumbar spinal cord, expressed in pmol of D-[^3^H]-aspartate transported per min and per mg of proteins. Transgenic rats only showed a higher uptake capacity at 21 days after surgery when compared to their sham control group ($ *p*< 0.05) and to the WT ligated group (# *p*< 0.05) on the ipsilateral **(A) **side. No changes were found on the contralateral **(B) **side. **(C, D) **Measure of the DHK-sensitive uptake activity in the ipsilateral **(C) **or the contralateral **(D) **dorsal horn of the lumbar spinal cord, expressed in pmol of D-[^3^H]-aspartate transported per min and per mg of proteins. **(C) **In the ipsilateral dorsal quadrant, the DHK-sensitive uptake was found highly increased in hSOD1^G93A ^rats at 21 days after ligation when compared to sham control rats ($$$ *p*< 0.001) or to WT ligated rats (### *p*< 0.001). The DHK-sensitive uptake was unaffected on the contralateral side **(D)**. Each group included 5 rats, ANOVA and Tukey post-hoc test as statistical analyses.

Quantitative measures of aspartate uptake in synaptosomes prepared from lumbar dorsal spinal cord samples were performed in order to evaluate changes in the activity of glutamate transporters. Consistent with the increased gene expression of the transporters, increased uptake was measured in ipsilateral samples from hSOD1^G93A ^ligated rats whereas no change was observed for WT ligated animals (Figure 6A). In the tested conditions, GLT-1-dependent uptake (estimated using the specific blocker DHK) accounted for approximately 25% of total uptake. The use of this blocker evidenced that DHK-sensitive uptake was statistically increased only in samples from hSOD1^G93A ^injured rats but not in WT ligated animals (Figure 6C). For both WT and hSOD1^G93A ^ligated groups, neither the total uptake nor the DHK-sensitive uptake were altered in samples from the contralateral dorsal horns (Figure 6B and D). Importantly, both gene expression and uptake capacities were found similar between WT and hSOD1^G93A ^sham groups.

### Increased gene expression of Nox2, IL-1β and TLR4 at 21 days after PSNL in hSOD1^G93A ^rats

**Figure 7:**
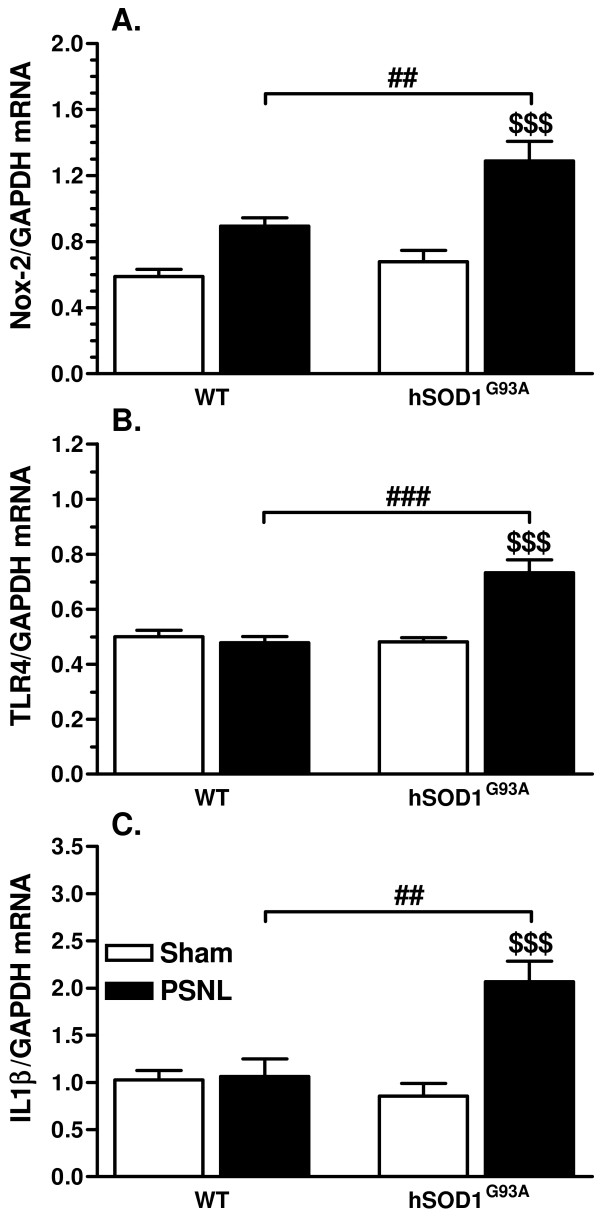
**hSOD1^G93A ^rats show increased gene expression of Nox2, TLR4 and IL-1β at 21 days after PSNL**. **(A) **mRNA levels of Nox2 (normalized for GAPDH mRNA expression) are higher in the ipsilateral dorsal quadrant of the lumbar spinal cord of transgenic rats at 21 days after PSNL, when compared to the other groups ($$$ *p*< 0.001; ## *p*< 0.01). **(B) **TLR4 gene expression was also increased in transgenic rats at 21 days after lesion when compared to their sham group ($$ *p*< 0.01) or the WT ligated group (## *p*< 0.01). (**C**) IL-1β transcript level was significantly higher in transgenic ligated rats at 21 days after lesion when compared to sham littermates ($$$ *p*< 0.001) or WT ligated rats (## *p*< 0.01). Each group included 5 rats, ANOVA and Tukey post-hoc test as statistical analyses.

To assess microglial activation, Nox2 mRNA (catalytic subunit gp91^phox^) [10] was quantified in the ipsilateral dorsal quadrant of the lumbar spinal cord at 21 days after surgery. While no difference could be detected between both sham groups, PSNL was shown to induce an increase in Nox2 gene expression. However, Nox2 mRNA up-regulation was found to be significant only for transgenic ligated rats when compared to their respective sham group, but also to WT ligated animals (Figure 7A). Because TLRs have previously been incriminated in neuropathic pain and TLR4 is the best characterized member of this family [9,32], TLR4 gene expression was measured in the ipsilateral dorsal quadrant of the lumbar spinal cord at 21 days after surgery. At this time-point, TLR4 mRNA levels appeared strongly increased in transgenic ligated rats as compared to the other groups. In contrast, no significant PSNL induced-increase in this pro-inflammatory receptor was detected in WT rats (Figure 7B). Pro-inflammatory cytokines are moreover clearly incriminated in neuropathic pain [33] and more precisely, IL-1β was shown to be a key cytokine in the development and maintenance of pain hypersensitivity [11]. Hence, gene expression of IL-1β was also quantified in the ipsilateral dorsal lumbar samples. Similarly, a substantial increase in IL-1β transcripts was only evidenced in transgenic animals after ligation when compared to the other groups (Figure 7C).

## Discussion

Microglial activation and subsequent neuroinflammatory/nitroxidative processes are known to directly influence neuropathic pain, however, such parameters were never studied after peripheral nerve injury in an animal model showing exacerbated inflammatory and nitroxidative properties, such as hSOD1^G93A ^rats. First, we were able to verify that at the age chosen, uninjured transgenic rats did not differ from WT rats in baseline pain behavior. Second, no differences were observed between both WT and transgenic sham groups for all biochemical parameters tested in the dorsal region of the lumbar spinal cord. The present study demonstrates that in transgenic rats expressing hSOD1^G93A^, PSNL leads to: (1)- increased microglial Iba1 up-regulation at early (day 3 post surgery) and late (day 21 post surgery) time-points; (2)- enhanced pain hypersensitivity; (3)- impaired astrogliosis at 21 days after lesion; (4)- increased expression and function of the glutamate transporters GLAST and GLT-1 at 21 days after injury; and (5)- increased gene expression of Nox2, TLR4 and IL-1β at 21 days after nerve injury.

### Inflammatory reactions are exacerbated in the dorsal spinal cord of hSOD1^G93A ^rats after peripheral nerve injury

Over-expression of hSOD1^G93A ^was already associated with inflammatory-prone properties in *in vitro *studies. Exposure of hSOD1^G93A^-derived glial cells to an activating stimulus indeed leads to enhanced production of pro-inflammatory mediators and increased protein carbonylation, as compared to activated glia from control animals [22-24]. We here provide the first evidence that hSOD1^G93A ^rats develop enhanced microglial activation upon peripheral nerve injury, as evidenced by enhanced Iba1 gene up-regulation as early as 3 days after PSNL. Moreover, the neuropathy-induced increase in Iba1 gene and protein expression was also found to be exacerbated in transgenic rats at 21 days after PSNL, reinforcing the statement that microglial activation in hSOD1^G93A ^rats is clearly amplified upon injury.

The underlying mechanisms of exacerbated microglial responses may originate from the activating mutation of hSOD1, causing an imbalance in the redox equilibrium that ultimately promotes the expression and release of pro-inflammatory mediators, as well as reactive oxygen/nitrogen species (ROS/RNS), after glial activation [22,23,34]. Importantly, similar Iba1 expression was measured for WT and hSOD1^G93A ^sham animals on postoperative day 3 (mRNA level) and 21 (mRNA and protein levels), implicating that exacerbated activation is not observed in the absence of lesion. Furthermore, it also highlights that at this early pre-symptomatic stage of ALS, there is no glial activation and no molecular changes in the dorsal region of the lumbar spinal cord, as previously reported [35]. The effects obtained after PSNL appear therefore independent of the development of ALS.

### hSOD1^G93A ^rats develop enhanced pain hypersensitivity after PSNL

In comparison with WT rats, hSOD1^G93A ^animals showed enhanced thermal hypersensitivity following PSNL, while the increase in mechanical hypersensitivity was only mild and did not reach statistical significance. This discrepancy may be explained by a biased floor effect in the assessment of mechanical sensitivity, as nerve injury induced-PWTs are typically low in WT animals. Alternatively, a differential mechanism may underlie thermal as opposed to mechanical hypersensitivity. It is indeed proposed that mechanical hypersensitivity requires a specific population of unmyelinated fibers [36] while thermal hypersensitivity is dependent on the expression of a specific protein in nociceptors [37]. Thermal and mechanical hypersensitivities were moreover shown to be regulated by different pathways in an aquaporin-4 KO animal model [38] or after pharmacological treatment with ROS scavengers [10].

While microglial activation is known to contribute to the development of neuropathic pain [3,39], it has also been suggested to participate in the maintenance of this hypersensitive condition [40]. Hence, the increased PSNL induced-hypersensitivity in transgenic rats may reflect the activation state adopted by microglia in the dorsal horn of the spinal cord. Indeed, different activation states of microglia have been documented, suggesting that their response is specialized and dictated by the nature of the stimulus [41]. Only some of these phenotypic changes have been directly linked to neuropathic pain and have been qualified as "pain-related" [8]. Hence, it may be proposed that the activation state reached by microglia after PSNL differs between WT and transgenic rats, with distinct consequences on the severity of pain symptoms. Indeed, hSOD1^G93A ^rats did not only show increased microglial activation, as evidenced with Iba1 gene and protein up-regulation, but also an increased gene expression of TLR4, which was absent in WT ligated rats. Because microglial TLR4 is linked to inflammation and pain [9], its high expression at late time-points supports the idea that hSOD1^G93A ^microglia adopt an exacerbated "pain-related" activation state after nerve injury. PSNL was moreover found to induce increased expression of the pro-inflammatory cytokine IL-1β in transgenic, but not in WT animals after 3 weeks. This cytokine was also clearly linked to neuropathic pain [42,43] and may therefore participate in the enhanced pain hypersensitivity observed in transgenic rats. Finally, ROS production associated with Nox2 activity was previously shown to be critical for pain hypersensitivity, but also for the production of pro-inflammatory mediators [10]. Because transgenic rats show enhanced Nox2 gene expression after PSNL as compared to WT ligated rats, we conclude that the increased ROS production together with the exacerbated inflammatory reactions may contribute to the enhanced pain hypersensitivity. However, we cannot exclude the possibility that beside glial cells, inherent changes in dorsal horn sensory neurons due to hSOD1^G93A ^expression, are to some extent involved in the increased pain effects.

Surprisingly, we did not observe mirror-pain after PSNL. Although PSNL was originally associated with mirror-pain [29], works from different laboratories frequently led to inconsistent results [44-46]. Interestingly, we observed a modest microglial Iba1 up-regulation in the contralateral dorsal horn of transgenic rats. This observation supports the concept dissociating microglial responses and mirror-pain in the PSNL model [44].

### hSOD1^G93A ^rats show impaired astroglial response in the spinal dorsal horn after PSNL

Consistent with previous investigations, PSNL induced an up-regulation of GFAP on the ipsilateral side of the spinal cord in WT rats [47,48]. However, such GFAP up-regulation appeared lower in hSOD1^G93A ^rats. Considering the increased lesion-associated hypersensitivity in these rats, this suggests that astrocytes do not exclusively exhibit pro-nociceptive effects in models of neuropathic pain. Indeed, it was already demonstrated that intrathecal grafting of PKC-activated astrocytes failed to induce pain behaviors [39]. Moreover, an inverse correlation has been reported between the intensity of mechanical hypersensitivity and GFAP immunoreactivity in the substantia gelatinosa at 3 months following complete sciatic nerve transection [49]. Hence, it cannot be excluded that the attenuated GFAP up-regulation in transgenic animals with PSNL may, to some extent, support the higher degree of neuropathic pain symptoms in these animals. The mechanisms involved in the attenuation of astroglial activation remain to be elucidated, but it is already known that the nature of cytokines is determinant in the resulting influence on astroglial reactivity [50]. Thus, it may be hypothesized that microglia, via the adopted activation state and the corresponding pattern of mediator expression, are able to differently drive astroglial responses. Of interest, IL-1β, which was clearly increased in transgenic rats at 21 days post PSNL, has previously been shown to decrease GFAP expression *in vitro *[51,52].

### Glutamate clearance system is improved in the dorsal horn of hSOD1^G93A ^animals after PSNL

Glutamate transporter expression is influenced by a variety of factors, particularly inflammatory mediators [13,14,53]. Altered expression of glutamate transporters was already evidenced in several CNS pathologies [54-56], as well as in neuropathic pain models [57-59]. Biphasic regulation of these transporters has been reported after nerve injury with an up-regulation in the first days and a down-regulation at 2 weeks [12,60,61]. Glutamate transporter down-regulation was suggested to enhance pain hypersensitivity due to impaired glutamate handling. We found that GLAST and GLT-1 were both up-regulated at 21 days after PSNL in transgenic rats and, accordingly, uptake activity was found to be increased. Although surprising at first glance, these findings might be related to differential expression in astroglia and microglia, as it was previously reported after PSNL [62]. Indeed, while in astrocytes, GLAST and GLT-1 immunoreactivity was decreased, microglia on the other hand showed a *de novo *synthesis of both glutamate transporters, in accordance with other *in vitro *[63,64] and *in vivo *[65] studies. The increased microglial expression of glutamate transporters may explain the overall increased uptake in transgenic ligated rats. Importantly, because transgenic rats showed increased pain hypersensitivity and microglial activation after PSNL, it suggests that microglia would not play a major role in regulating glutamate transmission.

## Conclusion

Even though increased processing of experimental nociceptive stimuli was recently evidenced in ALS patient [66], the study of pain in the context of ALS was out of the scope of the present research which essentially took advantage of the characteristic of an animal model of the disease at a pre-symptomatic stage. Thus, we here demonstrated that rats expressing hSOD1^G93A ^develop more severe pain hypersensitivity after peripheral nerve injury. Although this finding may be explained by a variety of cellular and molecular mechanisms, our data suggest the involvement of specific microglial activation states such as those characterized by TLR4 and Nox2 gene up-regulation, leading to an exacerbated inflammatory and nitroxidative environment. Moreover, sustained increases in IL-1β gene expression may also participate in the enhanced pain hypersensitivity. On the other hand, because astroglial activation was found to be attenuated in transgenic ligated rats, one may propose that activated astrocytes do not necessarily exert pro-nociceptive effects. Finally, the glutamate clearance system is affected in transgenic rats and this may additionally impact on the processing of pain signals in the spinal dorsal horn. The use of an animal model with SOD1 mutation showing more severe pain hypersensitivity and enhanced microglial activation after nerve injury may open new avenues in understanding neuropathic pain mechanisms.

## Competing interests

The authors declare that they have no competing interests.

## Authors' contributions

JVB was responsible for executing the entire research project, the statistical analyses and writing the manuscript. SG and SS assisted technically for rat breeding, aspartate uptake measurements and animal sacrifices. PL helped teaching the PSNL model. RD and EAJ supervised the immunohistochemical analyses and provided the material and facilities. EH directed the experiments and analyses and the writing of the manuscript. All authors read and approved the final manuscript.
